# Experimental Study on Vibration Fatigue Behavior of Aircraft Aluminum Alloy 7050

**DOI:** 10.3390/ma15217555

**Published:** 2022-10-27

**Authors:** Yunnan Teng, Liyang Xie, Hongyuan Zhang

**Affiliations:** Department of Mechanical Engineering and Automation, Northeastern University, Shenyang 110819, China

**Keywords:** aircraft aluminium alloy, material, vibration fatigue, experimental end criteria

## Abstract

It has been previously noted that the development of aerospace material technology and breakthroughs are inseparable when obtaining great achievements in the aerospace industry. Materials are the basis and precursor of modern high technology and industry. As one of the most powerful aluminium alloys, 7050 is widely used in the aerospace field. In this manuscript, the vibration fatigue behaviour of aircraft aluminium alloy 7050 is studied based on experiments. A vibration fatigue experiment and the traditional fatigue testing of aluminium alloy 7050 were performed. We found that there was an extreme difference between the vibration fatigue and the traditional fatigue curves. In addition, the experimental end criteria for the vibration fatigue experiment of aluminium alloy 7050 was obtained from the acceleration reduction and the frequency reduction value. For the acceleration experimental end criterion, 2% was the acceleration reduction value for the vibration fatigue experimental end criteria of aluminium alloy 7050. For the frequency experimental end criterion, 2% was the frequency reduction value for the vibration fatigue experimental end criteria of aluminium alloy 7050.

## 1. Introduction

In the aerospace field, many machineries, transportations and structural components have to be subjected to complex dynamic stress as the industry quickly develops [1,2,3,4,5,6,7,8]. What is most important is that lots of structures and mechanical components are constantly working in a vibrating state or even in the resonant condition, and this is particularly the case in the aeronautical field. In many conditions, the materials of aeronautical structures are subjected to vibration fatigue damages. The vibration fatigue behaviour of materials for aeronautical structures is a highly concerning topic for researchers. Many past damages [9,10,11] have proved that lots of structures are usually exposed to a vibrating state, nearly in the resonant condition, and this is especially true in aeronautical fields. Compared with traditional fatigue investigations, vibration fatigue analysis research deals with the material fatigue of flexible structures operated close to natural frequencies [12,13,14]. The essential difference in the mechanism of the vibration fatigue and the traditional fatigue is that vibration fatigue is affected by the dynamic stress parameters of the structures; thus, the influence of the structural dynamic characteristics should be considered.

Vibration fatigue is a kind of vibration-strength damage caused by vibration loads, and the damage is irreversible. More and more researchers have investigated the vibration fatigue performance of structural components in engineering practices, especially in the aeronautical field. Dentsoras and Dimarogonas [15] analysed the propagation rate of fatigue cracks for structures in resonant conditions. The investigation results showed that it was much quicker for the cracks to propagate in the resonance. With the propagation of the fatigue cracks, a local flexibility was introduced by the structures, which had an important effect on the dynamic responses. Damir [16] investigated the relationships between dynamic parameters and fatigue damages. By considering the modal parameters of the structures and the influence of structural dynamic characteristics on micro-structural deformation, the factors that influence vibration fatigue damages were studied. Concli, F. [17] studied the different fatigue criteria according to different results of crack propagation direction. Poursaeidi and Arhani [18] investigated the failure of an auxiliary steam turbine. Vibration fatigue damage problems [19,20,21,22,23,24,25,26,27,28] lead to serious structural failures, and their mechanisms are explored in investigations of the dynamic property of structures (such as natural frequency), random vibration problems and fatigue damage problems. In order to solve or avoid the problems caused by vibration fatigue, it is necessary to perform research on the vibration fatigue behaviour of structures. This approach was proposed by researchers to conduct a fatigue test on MZGS-100PL [29]. Based on the FPGA solution and using parallel technology, the fatigue test was performed on the NI CRIO-9074 controller. The results showed the benefits and high levels of application of the approach’s computing power, and that it is dynamically reconfigurable. Furthermore, Wojciech Macek [30] proposed a mixed mechanical metrological method to study the three-dimensional damages in specimens caused by the bending loading fatigue of the system. The results revealed the relationship between the accelerations and the fatigue damage, as well as the mentioned characteristic parameters of the system. Franco Concli [31] evaluated the accuracy of five different criteria by applying them to different fractured gear specimens. The results showed that the criteria of the mechanical components agreed with determining the most critical node for gear geometries. However, for a fatigue behaviour study on gears, the most appropriate criteria are the ones proposed by Findley and Papadopoulos. Murat Aykan [32] compared the fatigue damage of helicopter structural systems and evaluated the fatigue damage subjected to multi-axial loadings with vibration tests. The results led to the conclusion that the vibration simulation realism can be improved with multi-axis vibration testing. Sagi [33] estimated the fatigue life of a rotating shaft using Dirlik’s method and an experimental analysis. This approach to predicting the fatigue life was indicated as being useful for the rotating shafts. The AE method was proposed to detect fatigue damages early for ball-bearing mechanical systems [34]. Alan Hase compared the two approaches of using AE signal technology and vibration methods. The AE signal analysis was more appropriate than the vibration analysis to detect initial cracks of fatigue damage. Fansong Li [35] simulated the vibration stress and evaluated the strength in a non-stationary state based on structural dynamic theories. By comparing the experimental data, it was found that the simulation error was reasonable, and the proposed approach to vibration stress simulation was applied as an effective method for vibration fatigue stress simulation.

The purpose of this manuscript is to study the vibration fatigue behaviour of aircraft aluminium alloy 7050, and the experimental end criteria of vibration fatigue experiments is proposed. The vibration fatigue and the traditional fatigue S–N curves were obtained. The essential difference between vibration fatigue and traditional fatigue is that vibration fatigue involves the dynamic parameters of the structures; thus, the influence of the structural dynamic characteristics should be considered as F (ω, N). In addition, a key point of the vibration fatigue experiment is to make the experimental end criteria clear. Two experimental end criteria are proposed in this manuscript. For the acceleration experimental end criterion, 2% is the acceleration reduction value for the vibration fatigue experimental end criteria of aluminium alloy 7050. For the frequency experimental end criterion, 2% is the frequency reduction value for the vibration fatigue experimental end criteria of aluminium alloy 7050. The main body of this paper is split into four sections. The material specimen is presented in Section 2. The experiment is set up in Section 3. The experimental results are investigated in Section 4. In Section 5, important conclusions are summarized.

## 2. Material Specimen

**Figure 1:**
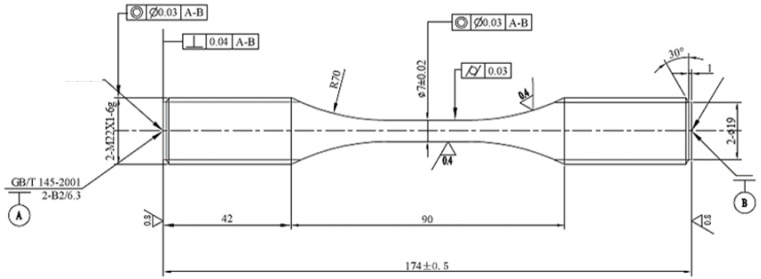
Dimensions and geometry of the specimen.

The aluminium alloy 7050 specimen used in the vibration fatigue test is shown in Figure 1. The geometry dimensions, chemical composition and mechanical properties of the specimen are listed in Table 1 and Table 2. Based on the testing reports of the manufacturer, the chemical composition of the alloy is as follows: Cu, 2.36%; Zr, 0.1%; Si, 0.12%; Fe, 0.15%; Cr, 0.04%; Mn, 0.1%; Zn, 5.7%; Mg, 2.3%; Ti, 0.06%; and the balance is Al.

## 3. Methods

**Figure 2:**
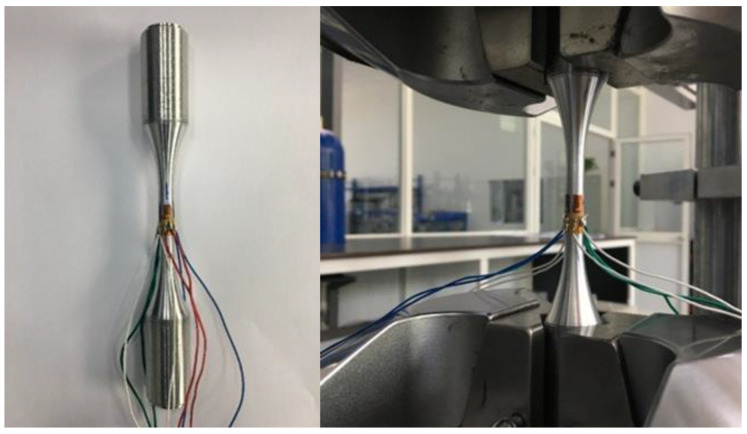
Vibration fatigue experiment platform.

The fatigue behaviour of the materials, characterized by the S–N curve (stress versus number of cycles), yields the number of cycles that occurred before the failure of the specimen depending on the amplitude of the stress applied. During vibration-induced failures, the frequency will shift as the crack grows through the material. The change in frequency leads to a change in the stress state. Experimental work is one of the most effective methods to study the vibration fatigue behaviour of materials. Moreover, it is the critical technique to ensure the vibration fatigue experimental end criteria for the vibration fatigue test. The vibration fatigue experiment and the traditional fatigue test were conducted. Firstly, we made the resonant frequency of the experimental system clear. Then, the specimen was swept with the frequency test before we conducted the vibration fatigue experiment. The vibration fatigue experiment was carried out at room temperature without heating throughout the whole process of testing, and the vibration fatigue experiment platform is shown in Figure 2.

To compare our study of the vibration fatigue with the traditional fatigue test, the traditional fatigue test was also performed. With the same experimental specimen of the vibration fatigue experiment, the specimen was used to perform the traditional fatigue test under the same condition and at the same temperature environment. Then, the experimental results of the traditional fatigue test were obtained.

**Figure 3:**
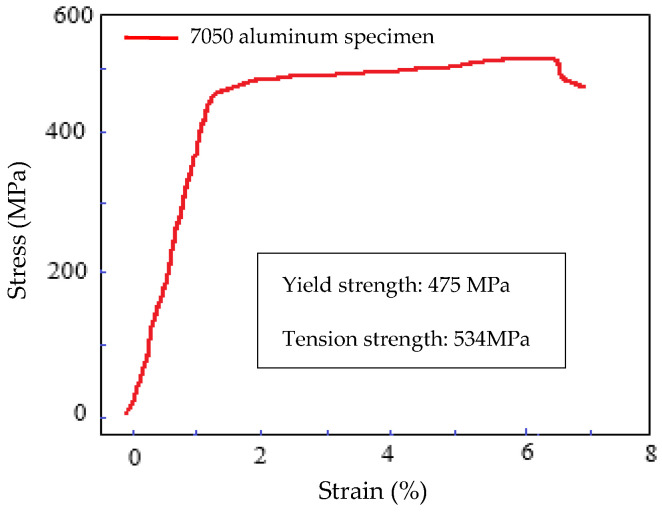
Stress–strain curve of aluminium alloy 7050.

The tension test and the acceleration response acquisition test were carried out to validate the stability and accuracy of the experimental system before fatigue testing. Then, we proceeded with the sweeping frequency test to confirm the natural frequency of the experimental system and the specimen of the vibration fatigue experiments. By using ASTM E8/E8M-15a, which is standard for the tensile test of materials, the tensile test of aluminium 7050 was carried out. The running speed was 0.005 mm/min before the yield. After that, the running speed became 5 mm/min. Then, the stress–strain curves were obtained as follows, which is shown in Figure 3.

**Figure 4:**
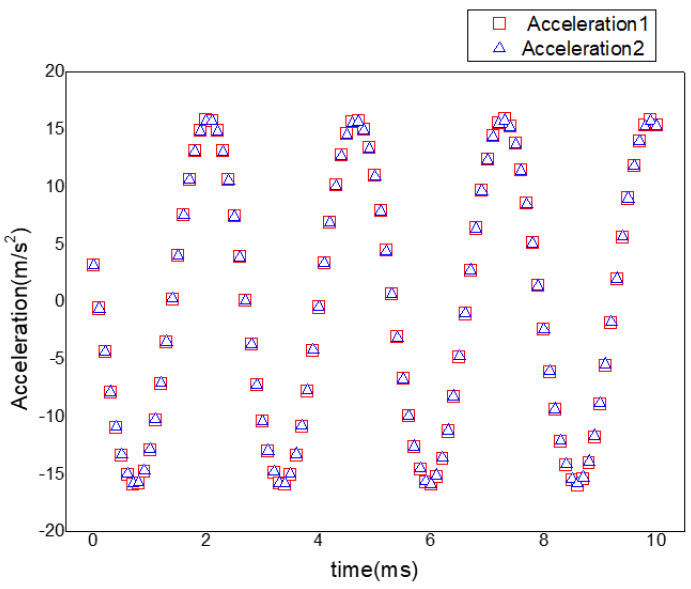
Acceleration response tests of aluminium alloy 7050.

The acceleration response test was performed with the electromagnetic shaking platform to validate the accuracy of the responses of the acceleration. Three acceleration sensors were used while testing. One was the acceleration sensor for inputting signals and the other two were the responses of the outputting signals. The amplitudes of the acceleration response curves were obtained approximately the same way. The maximum error was less than 1% and the trends of the curves were almost consistent, which is shown in Figure 4. Therefore, the testing results showed that the stability and accuracy of the testing system was reasonable.

## 4. Discussion and Results

**Figure 5:**
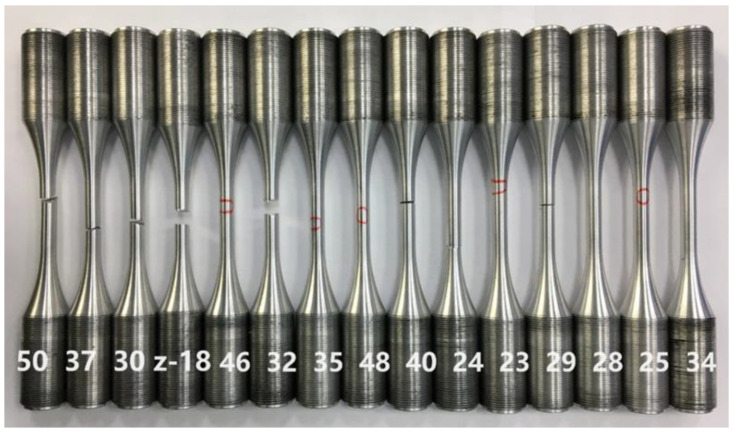
The specimen.

**Figure 6:**
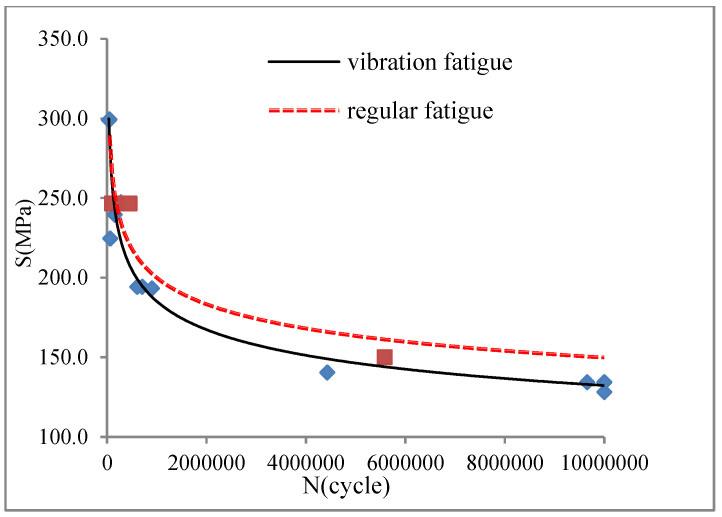
S–N curves of vibration fatigue and the regular fatigue.

The specimen used in the vibration fatigue experiment and the regular fatigue testing is shown in Figure 5. The vibration fatigue S–N curve and the regular fatigue S–N curve were both obtained. They are shown in Figure 6.

**Figure 7:**
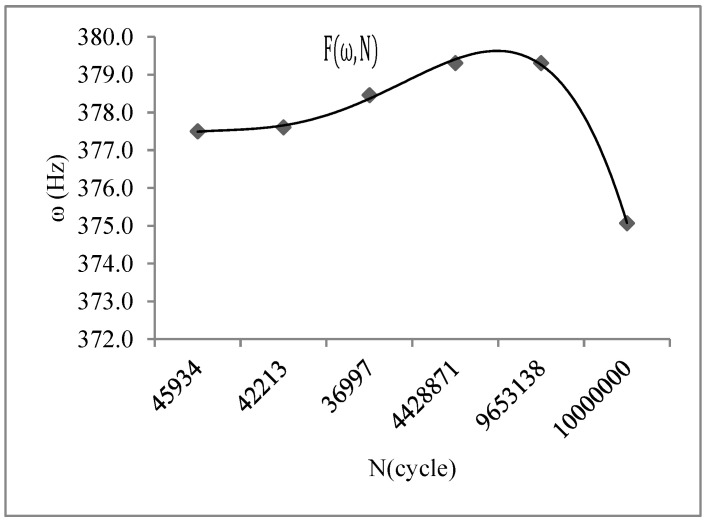
Vibration frequency–fatigue life of aluminium alloy 7050.

**Figure 8:**
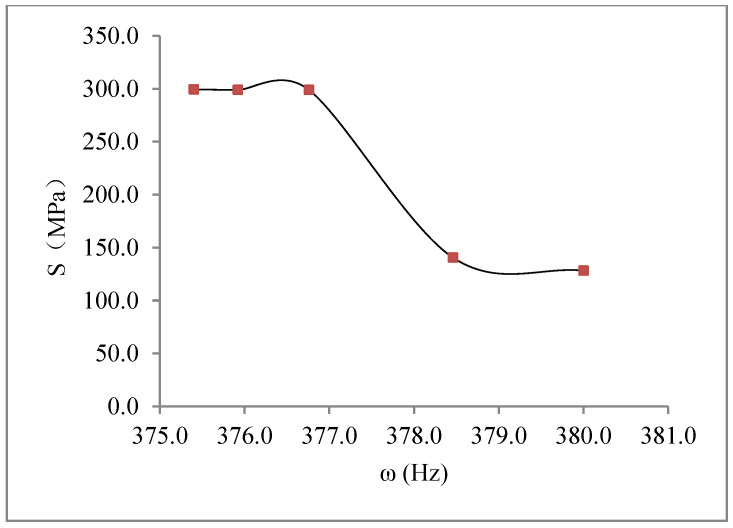
Vibration frequency–stress of aluminium alloy 7050.

Several valuable results were obtained with the vibration fatigue and traditional fatigue test. The S–N curve of the vibration fatigue showed exactly the same trends as the traditional fatigue S–N curve. However, there was an extremely different value between the curves. The fatigue strength of the vibration fatigue decreased compared with the regular fatigue strength. There was a difference in the value of the two curves. Based on the experimental data analysis, the corresponding relationship between the vibration frequency and the fatigue life was obtained as F (ω, N), and it is shown in Figure 7. With the vibration fatigue testing, the frequency slightly improved at first, and then it reduced rapidly. During fatigue testing, the frequency changed as the crack grew through the material. The frequency decreased with the crack propagation. Moreover, the relationship between the vibration frequency and stress was also obtained, and it is shown in Figure 8. The frequency changed the stress state of the structure. With the short increase in frequency, the stress was cut down and then became a constant.

**Figure 9:**
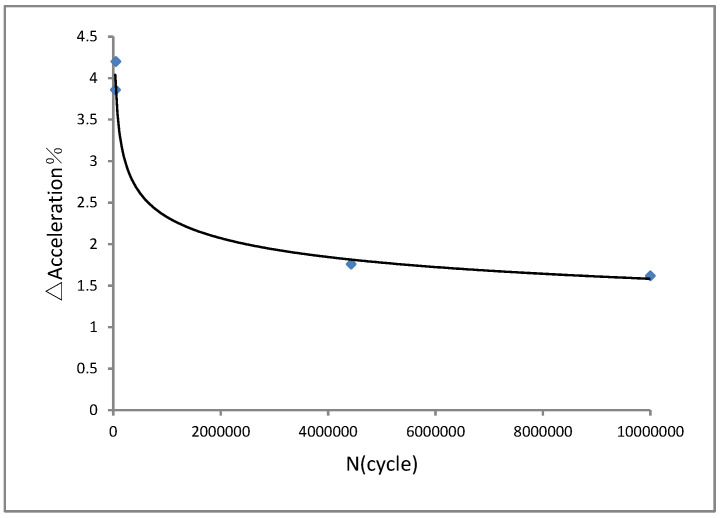
Acceleration reduction for vibration fatigue experimental end criteria.

**Figure 10:**
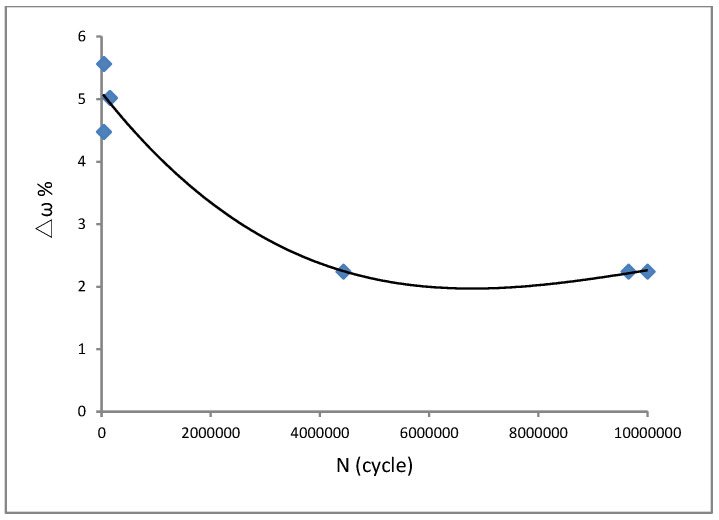
Frequency reduction for vibration fatigue experimental end criteria.

**Figure 11:**
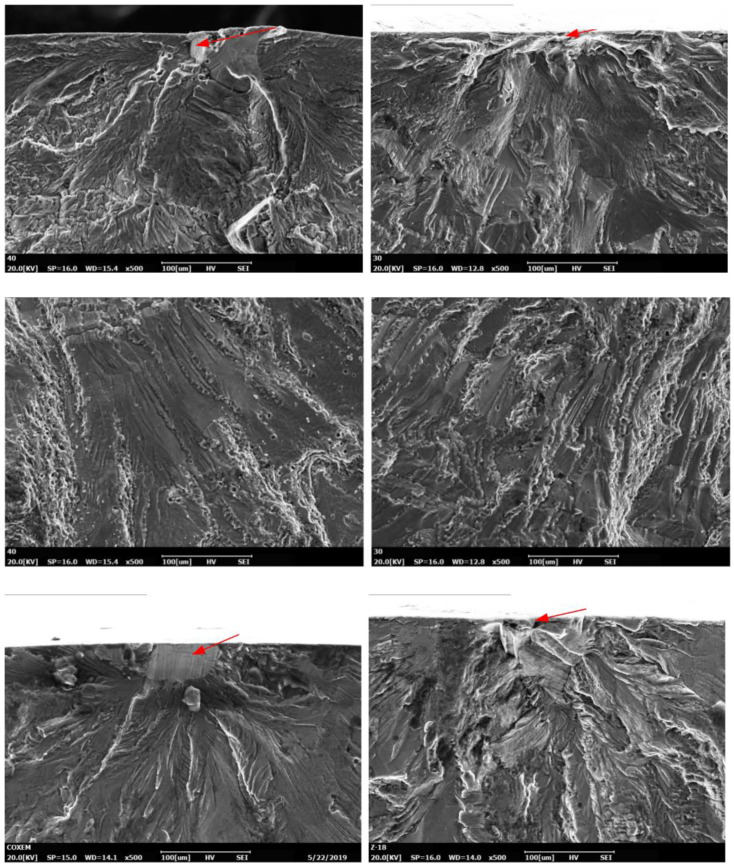

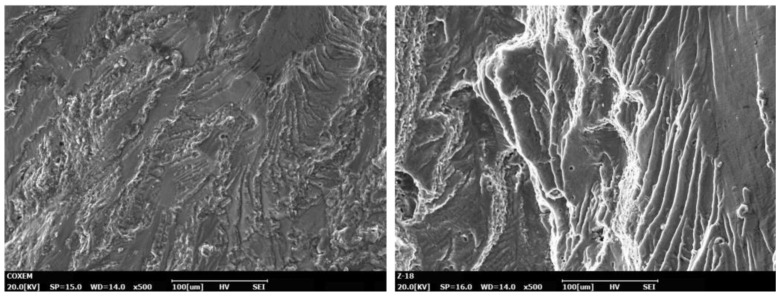
SEM images of vibration fatigue specimen.

In addition, the vibration fatigue experimental end criteria for the aluminium alloy 7050 was studied. The acceleration reduction in the vibration fatigue experiment of aluminium alloy 7050 is presented in Figure 9. We can see from the rule curve of the acceleration reduction in Figure 9 that 2% was the acceleration reduction deference value for the vibration fatigue experimental end criterion of the aluminium alloy 7050. The frequency reduction for the vibration fatigue experimental end criterion of aluminium alloy 7050 is shown in Figure 10. We found that 2% was the frequency reduction deference value for the vibration fatigue experimental end criterion of aluminium alloy 7050, as is shown in Figure 10. Furthermore, a fracture analysis was conducted on the vibration fatigue specimen using a scanning electron microscope. The results are shown in Figure 11.

The SEM images showed that the distribution of the grains in the materials was not homogeneous and not perfectly uniform in shape, and within each grain, the mechanical behaviour exhibited anisotropy due to the presence of grain boundaries. The presence of inhomogeneity was not only due to differences in grain structure, but also due to the presence of discontinuous regions in the material. Microscopic plastic deformation can occur at lower stress levels at the free surface. Crack sprouting is more likely to occur at the material surface or at defects such as inclusions and porosity due to the inhomogeneous stress distribution on the microscopic scale and the fact that the material surface grains are more conducive to cyclic slip. The source of the crack starts from the surface of the specimen. The microscopic morphology of the fracture shows a dissociated river-like pattern as well as slip features. It can be seen from the SEM images that the fracture features of the vibration fatigue specimen were almost consistent with the traditional fatigue. In the vibration fatigue test, the frequency changed due to the evolution of the structural damage. The frequency changed the stress state of the structure. It can be seen that the frequency was changed as the crack grew through the material. The frequency was decreased with the crack propagation.

## 5. Conclusions

In conclusion, the vibration fatigue behaviour of aircraft aluminium alloy 7050 was studied. Vibration fatigue and regular fatigue experiments were performed. The S–N curve of the vibration fatigue showed exactly the same trends as the regular fatigue S–N curve. However, there was an extremely different value between the two curves. The corresponding relationship between the vibration frequency and fatigue life was obtained as F (ω, N) for the aluminium alloy 7050. Furthermore, it is quite critical to ascertain the experimental end criteria for the vibration fatigue experiments of materials. The vibration fatigue experimental end criteria for the aluminium alloy 7050 was also studied. For acceleration reduction, 2% was the acceleration reduction deference value for the vibration fatigue experimental end criteria of aluminium alloy 7050. As other criteria for the vibration fatigue experimental end, 2% was the frequency reduction deference value for the vibration fatigue experimental end criteria of aluminium alloy 7050. In addition, a fracture analysis was performed with a scanning electron microscope on the vibration fatigue specimen. The microscopic morphology of the fracture phenomenon showed a dissociated river-like pattern as well as slip features. During vibration fatigue testing, the frequency changed due to the evolution of the structural damage. The frequency changed the stress state of the structure.

## Figures and Tables

**Table 1:** Dimensions of the specimen.

Description	Unit	Value
Working diameter	mm	6.98–7.02
Working length	mm	90
Density	kg/m^3^	2.8 × 10^3^

**Table 2:** Mechanical properties of aluminium alloy 7050.

Description	Unit	Value
Elastic modulus	Gpa	70
Ultimate tensile strength	MPa	534
Yield strength	MPa	475
Poisson	-	0.3

## Data Availability

Not applicable.
